# Modulating α-synuclein clearance: a comparison of high-frequency and direct current stimulation in preclinical models of Parkinson's disease

**DOI:** 10.1093/braincomms/fcag230

**Published:** 2026-06-23

**Authors:** Minesh Kapadia, Lorraine V Kalia, Suneil K Kalia

**Affiliations:** Krembil Research Institute, University Health Network, Toronto Western Hospital, Toronto, ON M5T 0S8, Canada; Krembil Research Institute, University Health Network, Toronto Western Hospital, Toronto, ON M5T 0S8, Canada; Division of Neurology, Department of Medicine, University of Toronto, Toronto, ON M5T 2S8, Canada; Tanz Centre for Research in Neurodegenerative Diseases, University of Toronto, Toronto, ON M5T 2S8, Canada; Krembil Research Institute, University Health Network, Toronto Western Hospital, Toronto, ON M5T 0S8, Canada; Division of Neurosurgery, Department of Surgery, University of Toronto, Toronto, ON M5T 2S8, Canada; Center for Advancing Neurotechnological Innovation to Application (CRANIA), Toronto, ON M5T 2S8, Canada

## Introduction

Parkinson’s disease involves degeneration of dopaminergic neurons in the substantia nigra pars compacta (SNpc). A defining pathological hallmark is misfolded α-synuclein (α-syn), which aggregates from monomers into oligomers, protofibrils, and insoluble fibrils that form dense Lewy bodies.^1^ Mutations in *SNCA* or increased expression of wild-type (WT) α-syn both promote aggregation and cause Parkinson’s disease. These toxic aggregates disrupt organelles, impair synapses, and drive neuronal death.^2^ Current treatment options, including dopaminergic pharmacotherapies and standard deep brain stimulation (DBS), provide symptomatic relief but do not stop or reverse neurodegeneration. Ongoing research is investigating whether enhancing α-syn clearance could serve as a disease-modifying strategy.^3^

Against this backdrop, we read with great interest the recent study by Bechkos *et al*.^4^ published in *Brain Communications*, showing that direct current stimulation (DCS) reduces α-syn accumulation in primary cortical neurons seeded with WT and mutant α-syn pre-formed fibrils (PFFs).^4^ DCS decreased aggregated and phosphorylated α-syn, including S129 phosphorylation linked to Parkinson’s disease pathology, while improving neuronal viability. We commend this work, which independently corroborates and extends our group’s recent findings that high-frequency electrical stimulation (HFS), mimicking DBS parameters used in Parkinson’s disease, reduces mutant α-syn expression and limits WT α-syn oligomerization in cultured neurons.^5^ Moreover, we showed that DBS delivered to the SNpc, but not the conventional subthalamic nucleus (STN) target, reduced α-syn levels in a preclinical model of Parkinson’s disease. Mechanistically, these effects appear transient and coincide with alleviation of α-syn-mediated dysfunction of protein degradation systems in cultured cells and cortical neurons.^6^

Here, we seek to contextualize the new DCS findings alongside our HFS and DBS results. By comparing these modalities, we highlight converging evidence that direct electrical stimulation is not only a neuromodulatory approach for symptomatic control, but also a potential disease-modifying therapy. We further examine how the timing of α-syn clearance provides insight into the mechanisms underlying these effects, including differential responses between WT and mutant *α*-syn.

## Converging evidence: efficacy across stimulation modalities and models

Both our findings and the recent report by Bechkos *et al*.^4^ validate electrical neuromodulation as a viable strategy to limit the accumulation and toxicity of α-syn. Crucially, this effect remains robust across different models of synucleinopathy. Bechkos *et al*.^4^ utilized exogenous WT and A53T PFFs to seed endogenous aggregation, modelling the prion-like cell-to-cell spread of Lewy-like pathology in mouse cortical neurons. In contrast, our group utilized adeno-associated virus (AAV)-mediated overexpression models to study the effects of human WT and A53T mutant α-syn in rat cortical neurons.^5^ This was paired with bimolecular protein complementation with split fluorescent protein reporters to detect and quantify α-syn oligomers *in vitro* and *in vivo*. Despite these different models, both approaches confirmed that electrical stimulation rescues primary cortical neurons from α-syn-mediated burden. Bechkos *et al*.^4^ also noted a modest increase in overall cellular viability following DCS, suggesting neurotrophic or protective benefits following depolarizing stimuli. Although we did not gauge cell viability following HFS, these findings align with our data that stimulation limits α-syn-induced neuronal dysfunction,^6^ and may thus prevent the acceleration of pathological cascades preceding neuronal death. Interestingly, assessment of tyrosine hydroxylase immunoreactive neurons yielded no differences following targeted DBS in a Parkinson’s disease rat model in which AAV vectors expressing α-syn fused to N- or C-terminal halves of YFP were directly co-injected in the SNpc.^5^ It is important to note that our study examined early, short-duration stimulation. In contrast, other studies have reported neuroprotective effects with prolonged DBS of the STN in an AAV-based A53T α-syn overexpression rat model,^7^ highlighting the need for future work to evaluate the impact of chronic stimulation paradigms.

## Temporal dynamics of α-synuclein clearance

The temporal dynamics of α-syn clearance reveal important distinctions between the different electrical stimulation modalities. Bechkos *et al*.^4^ found that a single, brief 30-minute session of DCS was sufficient to induce prolonged, delayed clearance. In their study utilizing ‘mature’ E17 cortical neurons, DCS-mediated reductions in intracellular aggregated and phosphorylated α-syn were not initially apparent 6 h post-stimulation, but were noted at the 48-h timepoint. Interestingly, the effects of DCS on α-syn were apparent 6 h post-stimulation in ‘immature’ E14 neurons. No direct comparisons were performed between the timepoints to assess kinetics of α-syn accumulation or phosphorylation, but the fact that α-syn was still cleared 48 h after a single DCS session suggests that the protein does not reaccumulate in neurons. We found that with HFS applied continuously for 3 h or a longer duration of 24 h, the reductions in both mutant α-syn and WT oligomers are successfully maintained in E17 neurons. In contrast to the need for continuous HFS, our follow-up study demonstrated that HFS applied for just 2 h yields a rapid, but transient reduction in mutant α-syn in cultured SH-SY5Y cells. Specifically, the therapeutic clearance was preserved 3 h post-stimulation but reverted to baseline non-stimulated levels after 24 h of recovery. Together, these findings highlight complementary temporal profiles, suggesting that DCS and HFS may engage partially distinct, yet potentially synergistic, mechanisms of α-syn clearance that differ in their onset and duration.

## Mechanisms and context-dependent nuances of α-synuclein clearance

While the outcomes of electrical stimulation converge, the two sets of studies interrogate contrasting mechanisms of α-syn clearance. Building on prior work on functional membrane depolarization in cultured SH-SY5Y cells,^8^ Bechkos *et al*.^4^ tested whether DCS promotes extracellular release of α-syn. Notably, they observed an early *decrease* in extracellular α-syn at 6 h post-stimulation only in the A53T PFF condition, with no effect in WT PFFs at this timepoint; by 24 h, reductions were evident in both WT and A53T conditions, before dissipating by 48 h. This effect was not mediated by exosomes, as levels of α-syn and ALIX, a canonical exosomal marker protein associated with ESCRT-dependent vesicle biogenesis, remained unchanged in isolated vesicles, suggesting instead the engagement of alternative intracellular degradation pathways. Consistent with this, we previously demonstrated that HFS-mediated reduction in transgene-derived mutant A53T α-syn is accompanied by restoration of protein degradation systems, without altering α-syn mRNA or endogenous α-syn protein levels.^6^ Specifically, HFS alleviates α-syn–induced impairment of the ubiquitin-proteasome and autophagy-lysosome pathways, including mitophagy, and may do so in part through modulation of the V-ATPase subunit ATP6V0C, promoting lysosomal re-acidification and enhanced protein clearance.

An important nuance across studies is the differential handling of WT versus mutant A53T α-syn. In our HFS model, reductions were robust for mutant A53T α-syn in cultured neurons, whereas total WT α-syn levels were unchanged, with effects restricted to *oligomeric* conformations of WT α-syn. In contrast, DCS more broadly reduced both WT and A53T α-syn, including aggregate-specific and phosphorylated forms regardless of the PFF type. It is plausible that different stimulation parameters (such as the 120 Hz, 0.4 ms pulse width HFS versus the 10 mV/mm, 1 ms pulse width DCS) preferentially recruit distinct, and potentially overlapping, clearance mechanisms with varying affinities for specific α-syn conformations (monomeric versus oligomeric or fibrillar α-syn) and sources (endogenous versus overexpressed), highlighting a context-dependent engagement of protein homeostasis pathways.

## Clinical translation: from in vitro to in vivo target specificity

As these electrical modalities move closer to clinical translation, the transition from *in vitro* to *in vivo* validation, and across the broader stimulation space, is paramount. While Bechkos *et al*.^4^ provide compelling *in vitro* data using cortical neurons, our 2024 work extended HFS into an *in vivo* rat model of DBS, demonstrating that anatomical targeting dictates therapeutic efficacy.^5^ Specifically, DBS targeting the SNpc reduced α-syn oligomers, whereas stimulation of the STN—a standard clinical target—did not alter α-syn levels or oligomerization. These findings underscore that optimal translation of HFS, DCS and other non-invasive modalities will require systematic refinement of anatomical targets, stimulation parameters (e.g. frequency, pulse width, current) and timing of intervention. Understanding how these parameters interact with disease-specific factors, including WT versus mutant α-syn, will be essential for tailoring interventions across sporadic and genetic forms of Parkinson’s disease.

## Conclusion

In conclusion, the findings presented by Bechkos *et al*.^4^ complement and extend prior work, expanding both the parameter space and mechanistic framework through which electrical stimulation may influence α-syn biology. While the concept of repurposing neuromodulation as a disease-modifying strategy is compelling, important questions remain regarding the magnitude and durability of α-syn reduction required for neuroprotection, potential thresholds of physiological α-syn depletion, and the optimal timing of intervention given that substantial dopaminergic neuron loss has already occurred at diagnosis. Encouragingly, preclinical data suggest that even modest reductions in α-syn oligomers, achieved by disrupting α-syn interactions with the ESCRT pathway, may mitigate neurodegeneration,^9^ supporting continued efforts to explore earlier intervention strategies. Together, these studies position electrical stimulation not only as a tool for circuit-level symptom control, but as a promising avenue for biologically informed, disease-modifying strategies in synucleinopathies and other neurological disorders^10^ warranting further mechanistic and translational investigation.
